# Antibiotic utilization pattern for surgical site infection prophylaxis at Dil Chora Referral Hospital Surgical Ward, Dire Dawa, Eastern Ethiopia

**DOI:** 10.1186/s13104-018-3629-6

**Published:** 2018-07-31

**Authors:** Yohanes Ayele, Henok Taye

**Affiliations:** 1 Department of Clinical Pharmacy, School of Pharmacy, College of Health and Medical Sciences, Haramaya University, P.O. Box 235, Harar, Ethiopia; 2 Dil Chora Referal Hospital, Dire Dawa, Ethiopia

**Keywords:** Surgical site infection, Surgical prophylaxis, Antibiotics

## Abstract

**Objective:**

The aim of this study was to assess utilization pattern of surgical antibiotic prophylaxis in surgical wards of Dil Chora Referral Hospital.

**Results:**

Prophylactic antibiotics were given in all surgical procedures. More than half of the participants 206(53.6%) were given Ceftriaxone while combination of Ceftriaxone and Metronidazole were used for 159(41.4%) patients. The most common procedure (88.3%), appendectomy, was managed with combination of Ceftriaxone and Metronidazole while the remaining was on Ceftriaxone. Hernia repair, another common procedure seen in this ward, was majorly managed by combination of Ceftriaxone and Metronidazole (60.7%) while the rest were on ceftriaxone alone. In general, inconsistence in antibiotic selection for different types of surgical procedures was seen. The surgical prophylactic antibiotics should be prescribed according to the international guidelines.

**Electronic supplementary material:**

The online version of this article (10.1186/s13104-018-3629-6) contains supplementary material, which is available to authorized users.

## Introduction

Surgical site infection (SSI) is defined as a proliferation of pathogenic microorganisms which develops in an incision site either within the skin and subcutaneous fat (superficial) and muscular facial layers (deep) or in an organ or cavity within 30 days after operation [1]. SSI is one of major complication of surgical procedures and represents a significant burden with regard to patient’s morbidity, mortality and hospital costs [2].

Surgical antibiotic prophylaxis (SAP) is administration of short course of antimicrobial agent prior to surgery to prevent SSI [3, 4]. There is ample evidence on the effectiveness SAP for prevention of SSI for most of surgical procedures [5–8]. However, for optimum prophylaxis, SAP should be used appropriately by considering the possible pathogen, pharmacokinetic of the drug, timing and route of administration, patient and procedure related factors [9].

Despite the evidence of the effectiveness of the appropriate use of surgical antimicrobial prophylaxis, its use is often found to be inappropriate. Report indicates that between 30 and 90% of this antibiotics prophylaxis use is inappropriate [10, 11]. Inappropriate use specifically in the area of the antimicrobial selection, timing and the duration of the antimicrobial prophylaxis were commonly observed [12–14]. Inappropriate usage could leads to increased hospital costs, emergence of resistant microorganisms, and super-infections and increased adverse drug reaction [15].

In developing countries, antimicrobials expense share higher budget compared to the other drug category. Antibiotics are one of the commonly used drug category in surgical procedures. However, regardless of its widespread use, very little is known about how these antimicrobials are being used particularly for prophylaxis of SSI and there were no similar studies conducted before in Dilchora Referral Hospital (DCRH). Therefore, the aim of this study is to assess utilization pattern of surgical antibiotic prophylaxis in surgical wards in DCRH.

## Main text

### Methods

This study was conducted at DCRH, Dire Dawa city administration, which is 526 km East of the capital of Ethiopia, Addis Ababa from January 2017 to June 2017. DCRH, the only referral hospital in the city, provides general outpatient, inpatient and emergency services for more than 45,000 populations in Dire Dawa administrative city population and nearby communities. According to 2016 annual report of the hospital, about 611 surgical procedures were performed in this hospital.

Facility based retrospective cross sectional study design was used to assess the utilization pattern of SAP in surgical wards of DCRH. Medications records of all patients admitted in surgery ward and who underwent surgical procedures were included in the study. In order to avoid difficulty in distinguishing prolonged prophylaxis from post-operative infection treatment, all dirty/infected wounds and those patients who had receive therapeutic antibiotic before the surgical procedure were excluded from the study. In addition, the documents that do not contain relevant information were excluded from the study.

Sample size was determined by using single population formula considering the P value of 50%, CI of 95% and marginal error of 5% giving total sample size of 384. The patient cards were selected by using systematic random sampling technique using calculated K-value (total clients in past 5 years divided by sample size). The data extraction format was developed and pre-tested and subsequently modified to ensure that the data would provide valid information. All relevant data was retrieved from patient’s medical records. Details of patient’s record including age, sex, admission diagnosis, type of surgery, wound class, and details of antibiotic prophylaxis including antibiotic choice, administration route, dosage form, dose and duration was retrieved and recorded (Additional file 1).

In the processing of data, each recorded data from the study participant’s card was labeled on the study participant’s checklist then the information found from the checklist format were cross checked by the data collectors and pharmacists in DCRH. The processed data was analyzed by using SPSS version 20 for compilation, summarization and comparisons of the numbers. Then frequency and percentage of the findings was calculated against each variable and the total study subjects.

### Results

#### Sociodemographic characteristics

A total of 384 patient cards were reviewed. The majority 159 (41.4%) of the patients were in age group of 31–65 years and just over half of the subjects 208 (51.2%) were male. Many of the subjects 228 (59.4%) were from urban areas and over one-third 148 (38.5%) had attended college and above while 115 (29.9%) were illiterate as shown in Table 1.

**Table 1:** Socio-demographic characteristic of study participants assessed for surgical site infection prophylaxis in Dil Chora Referral Hospital, Eastern Ethiopia 2017 (n = 384)

Variables	Number(%)
Age	
1–10	35(9.1)
11–30	153(39.8)
31–65	159(41.4)
Above 65	37(9.6)
Sex	
Male	208(54.2)
Female	176(45.8)
Religion	
Orthodox	153(39.8)
Muslim	180(46.9)
Protestant	51(13.3)
Residence	
Urban	228(59.4)
Rural	156(40.6)
Educational status	
Illiterate	115(29.9)
Primary	55(14.3)
Secondary	66(17.2)
College and above	148(38.5)

#### Medical and surgical history of patients underwent surgery

**Fig. 1:**
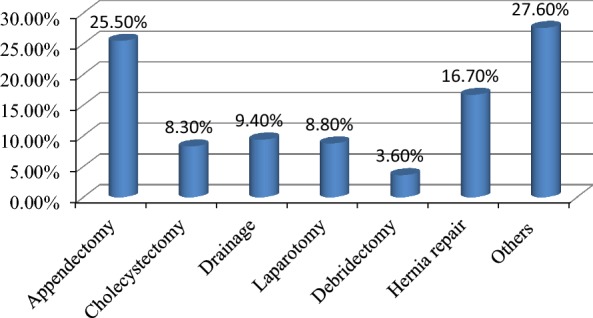
Type of surgical procedure Performed in Dil Chora Referral Hospital, Eastern Ethiopia, 2017 (n = 384). Others: Urology, head and neck, gynecology/obstetrics and vascular

Regarding the types of surgical procedure performed, appendectomy was leading procedure accounting about quarter (25.52%) of the procedure done in the ward followed by hernia repair (16.67%). Common procedures performed in DCRH are displayed in Fig. 1.

Concerning the types of wound, a large number of subjects 279 (72.7%) had clean surgery followed by 82 (21.4%) clean contaminated wound. The contaminated wound type was accounted a few 23(5.9%). Preoperative length of stay were also reviewed and just over half of subjects 195(50.8%) had emergency surgery while the remaining went through elective surgery.

#### Surgical antibiotics usage

All patients were given prophylactic antibiotics. Concerning commonly employed antibiotics, only two agents were administered for all types of surgical procedures, Ceftriaxone and Metronidazole. More than half of the participants 206(53.6%) were given Ceftriaxone while combination of Ceftriaxone and Metronidazole were used for 159(41.4%) patients. The remaining subjects were managed with Metronidazole alone.

**Fig. 2:**
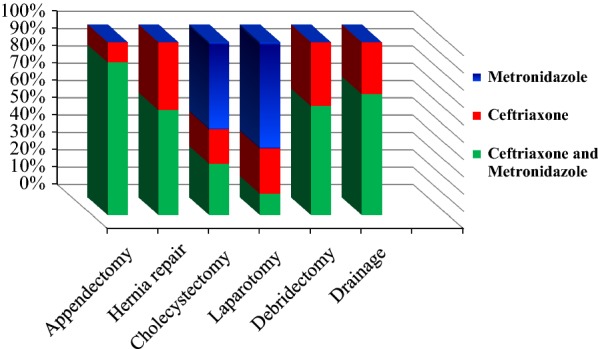
Type of surgical antibiotic prophylaxis used for commonly conducted surgical procedure in Dilchora Referral Hospital, 2017 (n = 384)

Regarding the selection of antibiotics for common procedures; most appendectomy procedures (88.3%) were given combination of Ceftriaxone and Metronidazole while the remaining was put on Ceftriaxone. Other common procedure seen in this ward, Hernia repair, was mainly managed by combination of Ceftriaxone and Metronidazole (60.7%) while the rest were put on ceftriaxone alone. In this ward, cholecystectomy was managed with different regimen; Metronidazole, combination of Ceftriaxone and Metronidazole and Ceftriaxone alone 49.3, 29.3 and 19.6% respectively (Fig. 2).

Concerning dosage and timing of administration of prophylactic antibiotics, a large number of patients 338 (88%) was given antibiotics 1 h before surgical procedures but the remaining were given just 30 min before the surgery. The majority 374(97.4%) of SAP was given as a single dose. Regarding the dose of ceftriaxone used in SAP, 1gm dose were used for 206(53.6%) patients, 2 gm were used for 30(7.5%). All patients who were put on Metronidazole were managed with 500 mg dose.

### Discussion

The primary objective of this study was to assess pattern of antimicrobial usage for surgical site infection prophylaxis. In present study, all patients who went through surgical procedure were given prophylactic antibiotics. Ceftriaxone and Metronidazole were only agents prescribed for all types of surgery, ceftriaxone alone being given for more than half (53.6%) of the patients.

In this study, all patients who went through surgery have given prophylactic antibiotics. Although this is finding is in line with study done elsewhere [16–18] it indicates overutilization of antibiotics in this ward. This might show lack of awareness and compliance toward international and local guidelines among health care providers.

International guideline promote the use less costly and narrow spectrum antibiotics for prophylaxis [19]. Cefazolin is widely indicated as first choice for most of surgical procedure. In our study however, over half of subjects were managed by ceftriaxone. Although this study is in line with study done in Addis Ababa where 70% of procedures were managed by Ceftriaxone [20], it is in contrast to Iranian [21] and Sudanes [17] studies in which they reported Cefazolin and Cefuroxime as the most commonly used prophylactic agent respectively. This may be related to inaccessibility of antibiotics and noncompliance toward international guidelines.

In present study, combination (ceftriaxone and metronidazole) were indicated regularly (41.4%). Although similar finding was reported in other study [20], the routine use combination seems inappropriate as such regimen is recommended only for limited procedures where anaerobic bacteria coverage is required [19].

In this study, inconsistences in antibiotics selection were seen. Different antibiotics were used for similar procedure. For instance, appendectomy was managed through combination of Ceftriaxone and Metronidazole (88.3%) as well as Ceftriaxone alone. According to the guidelines, anaerobic coverage is needed for appendectomy such as cephalosporin with anaerobic activity (Cefotetan) or Metronidazole combined with Cefazolin [19]. This kind of inconsistence and deviation from guideline recommendation were also observed in other studies [18].

Regarding dosage and timing of administration prophylactic antibiotics, in current study most of subjects (88%) were given antibiotics from half an hour to 1 h before surgical procedures indicating compliance to guidelines recommendation. This is similar with the finding of this study where all antibiotic prophylaxis were given within 30–60 min before surgery [22].

### Conclusion

It is concerning that in this ward all cases of surgical procedure were given antimicrobial prophylaxis either as single or combined forms. There was inappropriate antibiotic selection for different types of surgical procedure. The use of Ceftriaxone and combination of Ceftriaxone and Metronidazole for most procedure seems inappropriate.

#### Limitation of the study

The present study had limitation regarding its generalizability as it was not conducted in multicenter.

## Additional file

**Additional file 1.** The data collection tool is attached as a Additional file. It is the data extraction format used to collect data from patient records.

## Abbreviations

DCRH: Dil Chora Referral Hospital
SAP: surgical antibiotics prophylaxis
SSI: surgical site infection
