# Supplementary material for: Antibiotic utilization pattern for surgical site infection prophylaxis at Dil Chora Referral Hospital Surgical Ward, Dire Dawa, Eastern Ethiopia
Source: BMC Res Notes. 2018 Jul 31;11:537. doi: 10.1186/s13104-018-3629-6 (PMC6069961; doi:10.1186/s13104-018-3629-6)
Supplement: Supplementary file 1 — Additional file 1. The data collection tool is attached as a Additional file. It is the data extraction format used to collect data from patient records. [file 13104_2018_3629_MOESM1_ESM.docx]

**HARAMAYA UNIVERSITY**

**COLLEGE OF HEALTH AND MEDICAL SCIENCE**

**SCHOOL OF PHARMACY**

## Data Extraction format

This data extraction format is prepared to retrieve data from patient charts on the utilization pattern of antimicrobial surgical infections prophylaxis at DCRH surgical ward from January to June, 2017.

**1. Socio-Demographic Characteristics**

| S.No | Questions related with the respondent |  |  |
| --- | --- | --- | --- |
| 1.1 | Age | ______ |  |
| 2.2 | Sex | 1. Male  2. Female |  |
| 3.3 | Religion | 1. Orthodox  2. Muslim  3. Protestant  4. Others |  |
| 4 | Residence | 1.urban  2.rural |  |
| 5 | Educational status | 1.illiterate  2.primary  3.secondary  4.college/university level |  |

## 2. Medical and Surgical history of patients underwent surgery

| 1 | type of surgery | 1. emergency  2. elective |  |
| --- | --- | --- | --- |
| 2 | Type of surgical procedure performed for the patient | 1.Appedoctomy  2.Cholstoctomy  3.drainage  4.laprotomy  5.debirdectomy  6.Hernia repair  7.Other procedures_______ |  |
| 3 | Pre-operative length of time in minute and hour | __________ |  |
| 4 | Co- morbid condition do the patient has? | 1.DM  2. HIV-AIDS………..  3.TB  4.Hypertetion  5.Malignancy  6.Other co morbidity________ |  |

## 3. Surgical Antibiotics Usage

|  | Type of wound that the patient | 1.Clean  2.Clean contaminated  3.Contaminated  4.Dirty |  |
| --- | --- | --- | --- |
|  | Were any antibiotics employed for patient? | 1.Yes  2.No |  |
|  | If Yes, type of antibiotic given to the patient as prophylaxis | 1. Ceftriaxone 2. Ampcillin 3. Cefriaxone and Metrondazole 4. Metrondazole 5. Others------ |  |
|  | The form of antibiotics | 1.single  2.combined |  |
|  | Duration of treatment (in hours) | ----------- |  |
|  | Dose of antibiotics | ------------- |  |
|  | Dosage form of antibiotics | --------------- |  |
|  | Route of administration | 1.intera venous  2.intra muscular  3. intra dermal  3.other ------ |  |
